# Mirror life at the crossroads: Report on the symposia in Japan for discussion toward mirror life synthesis

**DOI:** 10.2142/biophysico.bppb-v23.0008

**Published:** 2026-02-20

**Authors:** Kei Fujiwara, Wataru Aoki, Chikara Furusawa, Ai Hasegawa, Gosuke Hayashi, Daisuke Kiga, Koichi Mikami, Sakura Takada, Yoshihiro Shimizu

**Affiliations:** 1 Department of Biosciences and Informatics, Keio University, Yokohama, Kanagawa 223-8522, Japan; 2 Department of Biotechnology, Graduate School of Engineering, Osaka University, Suita, Osaka 565-0871, Japan; 3 Center for Biosystem Dynamics Research, RIKEN, Kobe, Hyogo 650-0047, Japan; 4 Universal Biology Institute, The University of Tokyo, Tokyo 113-0033, Japan; 5 Department of Mechanical Engineering, Keio University, Yokohama, Kanagawa 223-8521, Japan; 6 Department of Biomolecular Engineering, Nagoya University, Nagoya, Aichi 464-8603, Japan; 7 Department of Electrical Engineering and Bioscience, Waseda University, Tokyo 162-8480, Japan; 8 Department of Foreign Languages and Liberal Arts, Keio University, Yokohama, Kanagawa 223-8521, Japan

## Abstract

The synthesis of mirror life, an organism composed entirely of the mirror-image counterparts of naturally occurring biomolecules, may be a topic for science fiction today but is suggested to be a reality in the future. Scientists called for strict preemptive regulation for the synthesis of mirror life due to concerns regarding biosecurity risks, such as the potential threat of mirror life to humans and non-human ecosystems. This perspective reports on the discussions held at two recent symposia in Japan, at the Japanese Society for Cell Synthesis Research (JSCSR) meeting and at the Biophysical Society of Japan (BSJ) annual meeting. Participants, including synthetic biologists, social scientists and artists, discussed the feasibility of the synthesis of mirror-image artificial self-replicating cells and the balance between the risks and benefits of this emerging technology. The discussions indicate that, while safety management is essential, a rational, evidence-based framework developed through open dialogue with society is crucial for the responsible advancement of mirror life research.

## Introduction

Most molecules that constitute all forms of life known to exist on the earth possess mirror-image isomers (enantiomers). Chirality exists not only in small molecules but also in the units of biomolecules. Proteins are polymers of L-amino acids, and DNA and RNA are polymers of 2-deoxy-D-ribose and D-ribose, respectively. Mirror-image life, of which all the constituents are enantiomers of the existing life, have frequently been depicted as an imaginary organism in science fiction (mirror-image life). However, recent advances in bottom-up synthetic biology have implied that mirror-image life is potentially an organism to be created in the future.

With the convergence of research on creating synthetic self-replicating cells by assembling biomolecules and advancements in technologies for the chemical synthesis of mirror-image biomolecules, many scientists in this field of science believe that the day we create mirror life in the laboratory will come in a few decades. However, facing the possibility of its happening, a considerable number of scientists have highlighted risks of mirror-image life, particularly of mirror-image bacteria, arguing that research involved in the synthesis of mirror life should be regulated [1,2].

For the production of knowledge and the emergence of new industries, one cannot ignore potential benefit of scientific research as well as its risks. This should also be the case for the research on the mirror life synthesis. Is mirror life really dangerous? Or does it represent a gateway to a new technology offering revolutionary value to our society? It is necessary to consider the appropriate form of regulation by weighing these multiple factors and inferring the characteristics of mirror life itself.

To address these questions from the standpoints of both science and society, two symposia were held in Japan. One is held at the Japanese Society for Cell Synthesis Research (JSCSR, a society aiming to provide a platform for discussion about the creation of synthetic self-replicating cells and technologies for *in vitro* synthetic biology), and the other is held at the Biophysical Society of Japan. In this perspective, we report on the talks and discussions that took place at these events and summarize a suggestion emerged from the symposia.

## Symposium on mirror life at 18^th^ meeting in Japanese Society for Cell Synthesis Research

JSCSR Meeting 18.0 was held at Rikkyo University in Tokyo, and we organized a symposium to discuss the regulation of mirror life in the afternoon on September 16th. First, Dr. Kei Fujiwara (Keio University), the organizer of this symposium, presented a review of the international discussion regarding the regulation of mirror life synthesis. Following this review, it was introduced that the authors of the Science opinion paper established Mirror Biology Dialogues Fund (MBDF) [3] to discuss this topic further.

Then, a video letter from Dr. James Smith (MDBF and J. Craig Venter Institute), who organized a series of international meetings on the mirror life synthesis as Deputy Director of MBDF, was presented with subtitles in Japanese (translated by Fujiwara). Smith mentioned that the results of an assessment of the risks associated with mirror life, which could potentially be created within the next 10 to 30 years, had been reported in Science [1]. It was explained that mirror versions of the existing bacteria (mirror bacteria) could have a profound impact on humanity once synthesized, due to the absence of natural predators and also because of the possibility that the immune system would not work on them. At the same time, it was explained that, in the Science paper, they argued that mirror life should not be created unless data to dispel concerns regarding its dangers is presented. It was further explained that the MBDF was established to continue discussion on mirror life, and one of the things it works on is assessing options to guard against these risks. In addition to the current call for global efforts to prevent the creation of mirror life, it was explained that various groups have recommended that research to create mirror bacteria should not proceed. It was also indicated that many have noted the importance of governance around enabling technologies that would be created to the pathway to making mirror life, to ensure that mirror life is not created.

Following this, Fujiwara explained the purpose of the symposium, stating that the goal was to deliberate on whether the regulation to prevent the creation of mirror life is truly necessary or not. It was explained that the reason he organized a symposium in this domestic meeting is that there are many researchers working in this field, as the PURE system, which is an essential big piece for realizing the creation of mirror life, was been developed in Japan.

According to this background, a video letter from Prof. Yoshihiro Shimizu (RIKEN), the original developer of the PURE system and a participant in the series of meetings organized by the MBDF, was projected (as he was attending an MBDF symposium being held in Manchester, UK, on that very day). Shimizu said that he has considered the synthesis of mirror life to be one of the goals of synthetic cell research. It was explained that the creation of mirror life could be achieved by the self-replicating PURE system, in which PURE system creates PURE system and conversion of the molecules of PURE system into their enantiomer. About the regulation of mirror life creation, Shimizu argued that unpredictability lies at the core of science and is also one of its most fascinating aspects. When a new technology emerges, researchers from different fields share new perspectives, which in turn give rise to further new research. Restricting a progress of particular science or technology is nothing more than closing off unpredictable future possibilities. Taking this into account, he suggested that research into the creation of mirror life is better to proceed, if containment to guarantee safety is possible.

Next, four speakers gave 10-minute talks to serve as “catalysts” for open discussion of the synthesis for mirror-image life to follow.

First, Prof. Wataru Aoki (The University of Osaka), an expert in biotechnology, discussed the benefits by the creation of mirror life. Aoki outlined how mirror nucleic acids, mirror proteins, mirror metabolites, and mirror biomaterials could contribute to human welfare. It was concluded that while discussions on the regulation are necessary, studies towards mirror life synthesis offers significant benefits; therefore, a facile prohibition would delay in obtaining such benefits.

Aoki predicted that the technologies required to synthesize mirror life would soon be available. It was pointed out that the importance of understanding the characteristics of mirror life to enable experiment in controlled environments before such life is created haphazardly. He also noted the possibility that excessive regulation could result in effects similar to those seen with the strict regulations on genetic modification. He emphasized that while it is easy to imagine risks, the understanding of benefits evolves only alongside technological innovation, making it difficult to balance the two regarding technologies that will be perfected in the future.

The second speaker was Prof. Ai Hasegawa (Keio University), a speculative design artist who provokes thought on ethical values, centered on themes regarding future desires as a course of their intersection with realistic technology. Hasegawa pointed out that the discussion on mirror life is analogous to recent trends in AI development. She highlighted the case of the “Pause Giant AI Experiments” open letter (March 2023) [4] from the Future of Life Institute, signed by Elon Musk and others. She noted that although a six-month moratorium was requested, development intensified regardless of the appeal. Also Hasegawa introduced a case study, Target Deception (2007) by the US group Critical Art Ensemble [5]. This performance critically verified the “politics of fear” by reversing the structure of a 1950 US military experiment, where harmless bacteria were released to simulate a biological warfare scenario.

Hasegawa said that she plans to release a work designed to facilitate understanding of the properties of mirror life. It was explained that she aims to create a piece that can be experienced in a short time, while carefully considering how to frame a “healthy discussion” that is neither mere scientific PR nor opposition, determining who should participate in the debate, and exploring how to think alongside the public.

The third speaker was Prof. Koichi Mikami (Keio University), who has a background in social science. First, Mikami explained potential difficulties with regulating the creation of mirror life. It was pointed out that, while it is necessary to define what constitutes research into the creation of mirror life, drawing the “red line” only for the related technologies is a challenge. Reflecting on the case of genome editing, where international regulatory landscapes varied, Mikami pointed out that establishing comprehensive global regulations has not been achieved. It was concluded that regulation in global research activities is challenging, noting also that from a bioeconomy perspective, there is a tendency for countries to adopt strategies that make their regulations as soft as possible to secure economic benefits.

Mikami also offered insights into how the discussion should proceed. Although international conferences for mirror life have involved hundreds of participants, it is still not enough to say that these discussions have sufficiently included a wide range of stakeholders. It was also pointed out that, while calling for regulation may appear a responsible action by scientists, without considering how to make it happen is equally irresponsible. It was emphasized that it is inappropriate for scientists to advance the discussion on their own because citizens are important stakeholders. Mikami invoked that the goal should be to realize open dialogue instead of hastily seeking for countermeasures to suggested risks.

The fourth speaker was Prof. Daisuke Kiga (Waseda University), a synthetic biologist who has led discussions involving ELSI (Ethical, Legal, and Social Issues) and RRI (Responsible Research and Innovation) on various occasions at the JSCSR meetings. Kiga indicated that examining the consequences of the creation of mirror life reveals the appropriate approach to its regulation, because new technologies always carry risks. It was also pointed out that there is a significant difference between “mirror artificial cells” and “mirror life”, and research up to the level of mirror artificial cells should be permissible. This view is based on the premise that artificial cells are extremely fragile, even if they possess self-replicating capabilities. However, it was also acknowledged that there is always the risk that they could be “upgraded”.

Kiga said that to prepare for the worst-case scenario, it might be necessary to stockpile mirror drugs and investigate whether mirror molecules can evade the immune system. It was also emphasized that the importance of accumulating experimental evidences on how mirror life affects existing organisms. Furthermore, he suggested that since a monitoring scheme for synthesis of genes of natural chirality is already in place, regulating synthesis of mirror genes that allows the upgrade of safe mirror artificial cells to dangerous mirror life would be the most feasible approach to take.

Following these ‘catalyst’ talks, a one-hour open discussion with the floor took place.

First, the discussion was on the current level of technology for synthetic self-replicating cells based on existing life. While it is widely believed that this will be realized within a few years to a decade, a leading scientist in this field (an attendee) suggested that even 100 years might not be sufficient for the synthesis of mirror life.

There was also a discussion regarding the “red lines” for research into the creation of mirror life, as well as a “whitelist” counterparts of research that can be conducted without issues. Regarding the red lines, the majority of researchers expressed resistance when assuming their own research might be included in it.

A comparison with regulations on genetic modification was also discussed. In the case of genetic modification, researchers appealed to the government to advance the technology amidst negative public reaction. In the case of mirror life, however, the situation is that scientists themselves are attempting to impose regulations. Under these circumstances, it is unlikely that citizens or the government would clamor for the research to proceed. Lessons from the past suggest that discussion is necessary precisely because the situation is reversed. Opinions were raised that the current problem is that regulations are about to be imposed without sufficient discussion, as Mikami suggested in his talk. A warning was issued that, as demonstrated by regulations on genetic modification, once regulations are introduced, it becomes difficult to remove them in the future; therefore, regulation of novel technologies like mirror life synthesis should be approached with caution.

Studies associated with human cloning were picked up as the rare example where an international ban has been successfully upheld. It was pointed out that while the use of human clones for organ transplantation was conceivable, the fact that its merits were limited when weighed against ethical considerations suggests that the benefits of mirror life should also be thoroughly investigated.

Citing the Dihydrogen Monoxide parody [6], it was noted that anything can appear dangerous if one focuses solely on potential hazards. An opinion was raised that if something is claimed to be dangerous, experiments demonstrating the real interaction between the matter and existing organisms are essential. As for another opinion, given that the definition of “life” is not fully established, the problem of conducting discussions using the vague term “mirror life” was also highlighted.

It was pointed out that if a system where the self-replicating mirror PURE system can be developed, many of the anticipated benefits could potentially be realized without the creation of full “mirror life.” There was also an opinion that precisely because it could pose a threat, it is necessary to keep it in a state where researchers can manage and study it.

A chief scientist at a synthetic biotechnology company shared a personal perspective: “From a purely commercial standpoint, if a mirror-image cell-free protein synthesis system is developed, it would likely be marketed as long as demand exists. However, the organization would likely take a cautious approach by restricting its availability to authorized clients.”

The possibility was also raised that mirror life might already exist in the earth but has been overlooked because current detection technologies are specialized for existing life forms. In relation to this, an example where samples collected from hydrothermal vents were cultured in a D-amino acid medium was introduced, but its result was the selection of existing life forms that expressed high levels of racemase [7].

A theoretical physicist studying the dynamics of life and death responded to the question of what would happen if a single mirror organism were to escape. His intuitive was that the probability of its survival would likely be close to zero.

To help citizens engage with the concept of mirror life, Fujiwara said that they published an introductory book, which combines five science fiction stories associated with mirror life and its synthesis with recorded dialogues between SF writers and scientists [8].

A web survey conducted among attendees revealed that some held the opinion that stricter regulations were necessary. However, both the floor discussion and the web survey made it clear that the majority opinion at this year’s JSCSR meeting was that there is no issue with the creation itself, provided the entities are controllable, such as fragile mirror artificial cells. They also agreed that rigorous management to contain mirror artificial cells in laboratory is essential.

## Symposium at the Biophysical Society of Japan

At the Biophysical Society of Japan, researchers from specialized fields delivered lectures and held discussions regarding the synthesis of mirror life. This symposium covered the possibilities, benefits, technical challenges, risks, and governance (regulations and social frameworks) for the synthesis of mirror life. The lectures proceeded from the potential for innovative applications brought about by mirror life (such as drug discovery and new materials) to the technical challenges required to realize them, practical examples of applications, evolutionary risks should mirror life be released into the environment, and finally, trends in international biosecurity and governance.

The first speaker was Prof. Aoki who also talked in the JSCSR meeting. Aoki introduced in vitro ribosome biogenesis and examined the advantages that would arise if mirror life were synthesized. Also, it was explained that utilizing the space of mirror-image isomers would effectively double the library size for biomolecules, leading to anticipated applications such as the discovery of new drugs via mirror metabolites, protease-resistant mirror antibodies, and mirror biomaterials (such as mirror silk). Estimates of the economic impact of these applications were also presented, and it was pointed out that the synthesis of mirror life would significantly accelerate research and development toward these.

The second speaker was Prof. Shimizu who sent the video letter for the JSCSR meeting. Shimizu introduced a history toward mirror life including the Pasteur’s famous enantiomer study [9], the synthesis of first mirror-image enzyme (D-HIV protease) [10], and mirror-image PCR and transcription [11–13]. Then, the international discussions regarding mirror life synthesis, such as the conferences in Asilomar, Paris, Manchester, and virtual ones were overviewed. It was pointed out that reconstitution of self-replicating cells from defined elements is indispensable for the synthesis of mirror life, and that constructing a mirror version of the PURE system is the key to achieving this. His ongoing work toward building a self-replicating PURE system was presented.

The third speaker was Prof. Gosuke Hayashi (Nagoya University). Hayashi reported on technologies for the selection of mirror antibody-like protein and the improvement of their functionality (binding affinity). Details were provided regarding the method for the chemical synthesis of mirror-image proteins and in vitro selection to find their binder. It was shown that although the products of mirror-image display initially possessed weak affinity, their binding affinities can be improved through affinity maturation, ultimately resulting in binder proteins maintaining a very high affinity of 0.2 nM. This result strongly supported the notion that mirror image protein binders (D-proteins) can recognize L-proteins with high affinity.

The fourth speaker was Prof. Chikara Furusawa (RIKEN and The University of Tokyo), an expert in evolutionary biology of microbes. Based on findings from evolution experiments for bacteria, Furusawa explained that evolution has preferred directions and is constrained by insurmountable barriers. Furthermore, utilizing these insights, he discussed the risks associated with the leakage of mirror bacteria into the environment. It was stated that the potential for proliferation would likely be determined by the balance between low fitness caused by nutrient shortage and the absence of natural predators such as phages. Additionally, Furusawa pointed out that mirror life cannot engage in genetic interactions (horizontal gene transfer) with natural life. Without genetic interactions, it has been shown that evolution involving drastic changes referred to as “jumps” is rarely to occur. Assuming a slow rate of evolution, it was estimated that even if mirror life with a genome lacking synthesis systems for complex compounds like biotin or tryptophan were released into the wild, it would be extremely difficult for it to adapt by acquiring the missing biosynthetic pathways through evolution before their extinction. Furthermore, comparing this to existing organisms and methods for their modification, he pointed out the possibility that the global threat posed by the synthesis of mirror life is relatively low.

The fifth speaker was Prof. Makiko Matsuo (The University of Tokyo), an expert in policy, ELSI and RRI. Matsuo introduced the fact that discussions on risks and examinations of policy options related to mirror life have already begun overseas, particularly from the perspective of biosafety and biosecurity. It was stated that because society is also a stakeholder, responsible research conduct in mirror life research is required, and the risks and concerns to the human health and the environment that have already been raised by scientists must be taken into consideration. It was also explained that if we are willing to realize the synthesis of mirror life, it is necessary to obtain scientific evidence demonstrating that there are no issues as ensuring safety is a prerequisite for the introduction of any emerging technology into society. Furthermore, it was pointed out that given that there are numerous emerging technologies not limited to mirror life, it is essential to address risks in proportion to the level of risk they pose. To achieve this, Matsuo noted that policymakers and funding agencies should provide support and allocate resources for research that strengthens safety, security, and governance in parallel with technological development.

After the symposium with short but significant discussions, an opinion was raised that “excessive regulation of mirror life synthesis could lead to research stagnation, similar to what happened with genetic modification.” It was proposed that “mirror life (including self-replicating artificial cells)” and “mirror biological systems (like PURE system)” should be clearly distinguished during discussions. The importance of securing the funding in order to gather scientific evidence as a prerequisite for risk assessment was emphasized in advancing research.

## Conclusion

Through these two symposia, it was suggested that the content of discussions regarding the synthesis of mirror life can vary significantly depending on their starting point. This is shown by the fact that different conclusions are reached based on the initial premise: the symposium in JSCSR meeting started from the perspective of whether regulation is actually necessary; that in the BSJ meeting started by questioning the probability of a crisis caused by mirror life; whereas MBDF emerged after a technical assessment of risks associated with mirror life and has supported broader expert scrutiny of the assessment, while also considering governance options for preventing the synthesis of mirror life.

Admittedly, the possibility cannot be denied that this stance, which many expressed that they are not necessarily negative toward the creation of mirror life is based on the characteristics of the attendees in the symposia, is based on the characteristics of the attendees in the symposia. However, it should be noted that opposing opinions were raised by researchers outside the symposia in Japan even at the eLetters and comments to the Science paper [14,15]. One commentary emphasizes the need for a balanced approach, stating: “*Given the countless unanswered questions, careful consideration of the scientific facts learnt so far [...] is crucial for bridging divergent views and fostering rational and informed debate*” [16]. It should also be noted that various reports and statements have reported findings and recommendations broadly consistent with the concerns summarized in the Science paper [17–21]. Moving forward, it is considered crucial to accumulate pieces of evidence in experimentation to determine whether mirror life is truly dangerous, the extent of that danger, and whether the benefits brought by mirror life are truly outweighed by the threats. It is essential to address these points and consider the ideal form of regulation while engaging society in the process.

## Acknowledgement

We would like to thank Prof. Makiko Matsuo (The University of Tokyo) for critical reading and comments on this manuscript. The symposia introduced in this commentary is supported by the Ishii-Ishibashi Fund (The Keio University Grant for Early Career Researchers).
